# Interaction of RAS Activation and Lipid Disorders Accelerates the Progression of Glomerulosclerosis: Erratum

**DOI:** 10.7150/ijms.108559

**Published:** 2025-02-28

**Authors:** Kun-Ling Ma, Jie Ni, Chang-Xian Wang, Jing Liu, Yang Zhang, Yu Wu, Lin-Li Lv, Xiong-Zhong Ruan, Bi-Cheng Liu

**Affiliations:** 1 Institute of Nephrology, Zhong Da Hospital, Southeast University School of Medicine, Nanjing City, Jiangsu Province, P.R. China.; 2 Department of Infection Management, Zhong Da Hospital, Southeast University School of Medicine, Nanjing City, Jiangsu Province, P.R. China.; 3 Centre for Nephrology, University College London (UCL) Medical School, Royal Free Campus, UK.

When reviewing our previous work, we realized that the picture of Oil Red O staining in human renal mesangial cells (HMCs) incubated with 10^-7^ mol/L Ang II presented in Fig. 1A (Group Ang II) was incorrectly assembled. The picture was misused by accident at that time. We are apologized to our readers for this error. After checking the original data, we found the original image and updated it into Figure 1A. All the authors have confirmed that the correction made in this erratum does not affect the original conclusions.

In addition, we found another error where the text on the vertical axis of Figure 1B is garbled. We checked the original text and found no problem. It is estimated that the garbled text could be caused by formatting during publication. We have corrected it in Figure.1B.

## Figures and Tables

**Figure 1:**
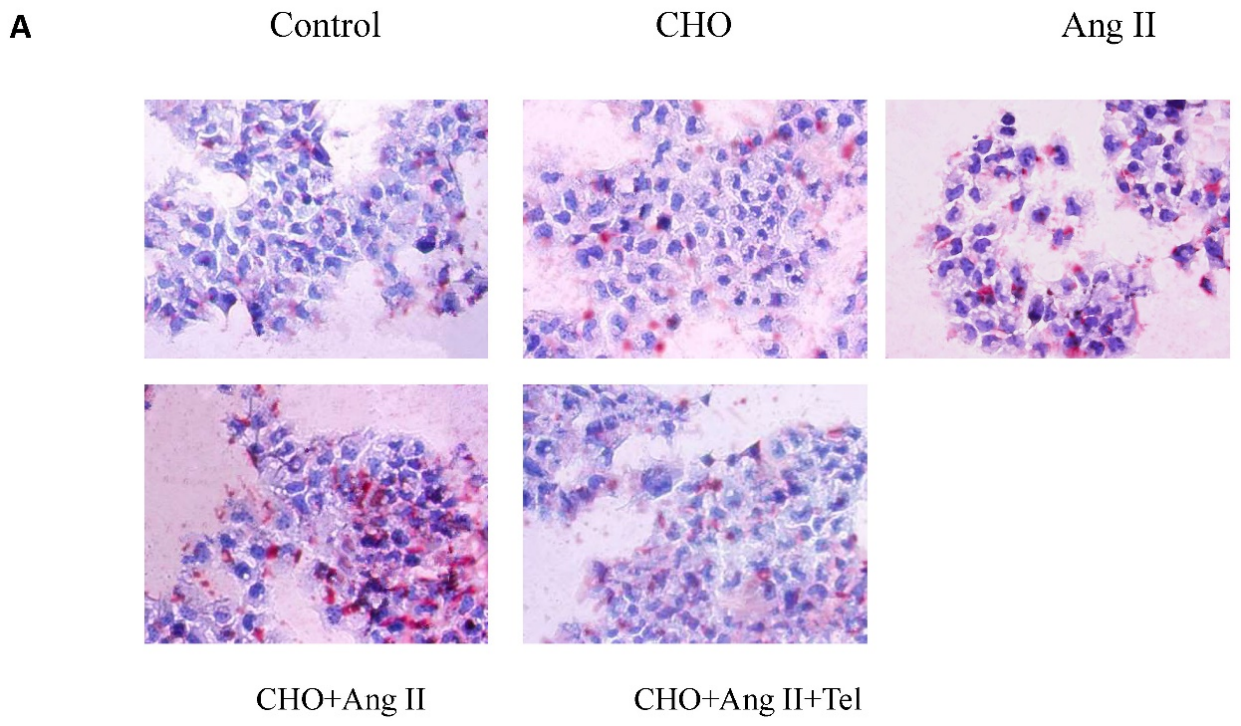

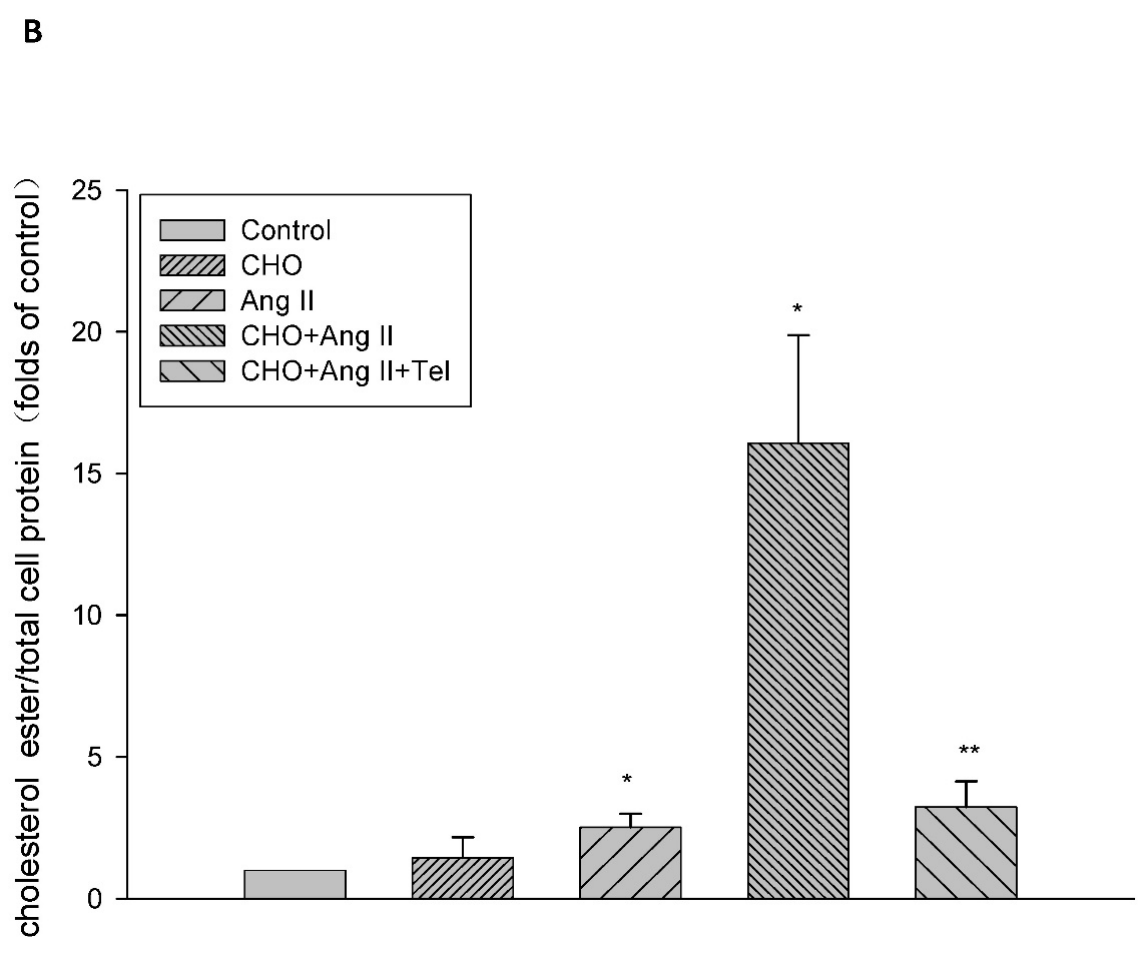
** Visualization of lipid accumulation in HMCs treated with Ang II.** HMCs were incubated for 24 hours in a serum-free medium in the absence (Control) or presence of 30 μg/mL cholesterol (CHO), or 10^-7^ mol/L Ang II (Ang II), or 30 μg/mL cholesterol + 10^-7^ mol/L Ang II (CHO+Ang II), or 30 μg/mL cholesterol + 10^-7^ mol/L Ang II + 10^-6^ mol/L telmisartan (CHO+Ang II+Tel). (A) The cells were examined for lipid accumulation by Oil Red O staining. The results are typical of those observed in four separate experiments (magnification ×200). (B) Effects of Ang II on intracellular free cholesterol and cholesterol ester in HMCs. Intracellular free cholesterol and cholesterol ester were assayed as described in Materials and Methods. Values are expressed as the mean ± SD of triplicate wells from four experiments. *P<0.05 *vs*. control, **P<0.01 *vs* CHO+Ang II.

